# Cushing's Disease Presented by Reversible Dilated Cardiomyopathy

**DOI:** 10.1155/2015/980897

**Published:** 2015-11-16

**Authors:** Berna İmge Aydoğan, Demet Menekşe Gerede, Asena Gökçay Canpolat, Murat Faik Erdoğan

**Affiliations:** ^1^Department of Endocrinology and Metabolism, Ankara University Faculty of Medicine, 06410 Ankara, Turkey; ^2^Department of Cardiology, Ankara University Faculty of Medicine, 06410 Ankara, Turkey

## Abstract

*Introduction*. Dilated cardiomyopathy is rarely reported among CS patients especially without hypertension and left ventricular hypertrophy.* Materials and Methods*. We hereby report a Cushing's syndrome case presenting with dilated cardiomyopathy.* Results*. A 48-year-old female patient was admitted to our clinic with severe proximal myopathy and dilated cardiomyopathy without ventricular hypertrophy. Cushing's disease was diagnosed and magnetic-resonance imaging of the pituitary gland revealed a microadenoma. Under diuretic and ketoconazole treatments, she underwent a successful transnasal/transsphenoidal adenomectomy procedure. Full recovery of symptoms and echocardiographic features was achieved after six months of surgery.* Conclusion*. Cushing's syndrome must be kept in mind as a reversible cause of dilated cardiomyopathy. Recovery of cardiomyopathy is achieved with successful surgery.

## 1. Introduction

Cushing's syndrome (CS) is a result of hypercortisolism due to adrenocorticotrophic hormone (ACTH) producing pituitary/ectopic tumors or cortisol synthesizing adrenal tumors. Besides many characteristic features, cardiovascular system may also be affected by disease and cardiovascular involvement is a major cause of mortality.

Hypertension is the most frequent cardiovascular complication of CS and provokes heart failure and ischemic heart disease. On the other side, dilated cardiomyopathy is rarely reported among CS patients especially without hypertension and left ventricular hypertrophy. We hereby report a Cushing's disease patient presenting with severe proximal myopathy and dilated cardiomyopathy.

## 2. Case Report

A 48-year-old female patient was admitted to our Department of Endocrinology with facial plethora, dyspnea, palpitation, edema, and severe proximal muscle weakness. She had a history of 10 kg weight gain and oligomenorrhea in the past three years. Type 2 Diabetes Mellitus was diagnosed two years ago and she was receiving long-acting insulin therapy in low doses. Glycohemoglobin level was 6.8%.

On physical examination, her blood pressure was 130/90 mmHg and pulse rate was 110 beats per minute. She had central obesity (body mass index: 27 kg/m^2^, waist circumference: 97 cm), buffalo hump, and abdominal purple-blue wide striae.

The physical examination of cardiovascular system was performed on admission. She had signs of heart failure. S3 gallop was heard on cardiac auscultation. The rales were found at the basal area of lungs. Bilateral peripheral edema was seen. The jugular venous pressure was 11 cmH_2_O.

Her electrocardiography just revealed sinus tachycardia on admission and there were no ischemic changes or left ventricular hypertrophy.

On laboratory examination, complete blood count and cardiac enzymes were within the normal ranges (Table 1). Liver function tests were mildly elevated but returned to normal during follow-up. On the basis of clinical features, Cushing's syndrome was the suspected diagnosis. Evaluation of plasma cortisol secretion showed absence of circadian rhythm. Twenty-four-hour urinary free cortisol level was high. No cortisol suppression was assessed after overnight 1 mg and subsequent 2 mg (classical two days) dexamethasone tests. Hormonal evaluation indicated ACTH-dependent Cushing's disease (Table 1).

Transthoracic echocardiography revealed dilated left ventricle [left ventricle end-diastolic diameter of 3.77 cm/m^2^ (normal range < 3.2 cm/m^2^) and left ventricle end-systolic diameter of 2.91 cm/m^2^ (normal range < 1.9 cm/m^2^) on M-mode] and ejection fraction was 20% (normal: 50–70%). All left ventricle (LV) wall segments were severely hypokinetic except posterior and lateral basal walls. Minimal pericardial effusion (0.6 cm behind left ventricle during diastole), minimal tricuspid, and mitral regurgitation were also noted. The thickness of interventricular septum and posterior wall was measured as 1 cm. There was no left ventricular hypertrophy (Table 2).

Angiotensin converting enzyme inhibitor and furosemide treatment were started with the diagnosis of decompensated heart failure, due to dilated cardiomyopathy. After clinical improvement of heart failure, beta blocker treatment, metoprolol succinate at 25 mg dose, was started.

**Figure 1:**
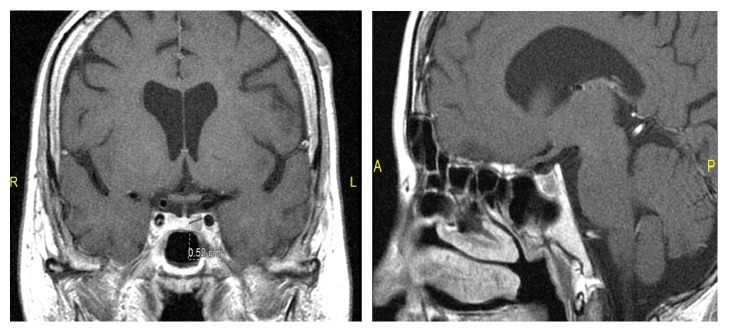
Coronal and sagittal T1-weighted image, showing a 5 mm microadenoma at the left side of pituitary gland.

Magnetic-resonance imaging of the pituitary gland revealed a 5 × 5 × 4 mm microadenoma located in the left side of the pituitary gland (Figure 1). Contrast enhanced thorax-abdomen CT was normal. The diagnosis was pituitary dependent Cushing's syndrome.

Surgical procedure was delayed because of the high risk of general anesthesia. Ketoconazole 800 mg/day was used for the preparation before surgery. Medical treatment was continued for three months. Although ejection fraction of left ventricle was still 30% on echocardiography (Table 2), improvement of heart failure symptoms was achieved (New York Heart Association (NYHA) class II). The coronary angiography at this stage excluded ischemic heart disease with normal coronary arteries.

She underwent a successful transnasal/transsphenoidal adenomectomy procedure. Clinical and laboratory findings improved significantly soon after surgery and she achieved complete remission of CD (Table 1). Also she was normoglycemic without medications and glycohemoglobin A1C was 5.5%. The patient was in NYHA class I. On transthoracic echocardiography, left ventricle end-diastolic diameter was 3.1 cm/m^2^, left ventricle end-systolic diameter was 1.7 cm/m^2^, and ejection fraction was calculated as 51%. There was a significant improvement in the left ventricular wall motion. The left ventricular wall thickness was measured within the normal range (Table 2).

## 3. Discussion

We report a case with dilated cardiomyopathy (DC) as the presenting feature of Cushing's disease (CD). Cardiovascular complications of Cushing's disease are attributable to metabolic syndrome components caused by hypercortisolism. Arterial hypertension is very common among these patients and reported to have 75% frequency [1]. Usually hypertension results with left ventricle hypertrophy (LVH), cardiomegaly, diastolic dysfunction, and ischemic heart disease [2, 3]. Dilated cardiomyopathy is rarely reported in these patients. We could find only seven previous reports of reversible DC in CD and pituitary adenoma was the etiological factor in three of them [4–6]. Excess lipid deposition in heart is one of the other suggested mechanisms that may lead to dilated cardiomyopathy and cardiac dysfunction [7].

It was also reported that LVH may also occur in normotensive Cushing's syndrome patients [8] and thus, insulin resistance, dyslipidemia, and truncal obesity also contribute to cardiovascular disease process in these patients. Successful treatment of Cushing's disease provides improvement in myocardial function independent of metabolic changes [9]. Impairment of both systolic and diastolic functions in active Cushing's disease was demonstrated by echocardiography and tissue Doppler studies [10]. Thus, potential mechanisms that affect cardiac status in hypercortisolemic state are questionable. Direct effects of hypercortisolism on myocardial tissue were defined formerly [11]. Cortisol leads to increased catecholamine response in myocardium and activates renin-angiotensin axis [12]. Mihailidou et al. demonstrated that cardiac damage is aggravated by activation of mineralocorticoid receptors by cortisol or glucocorticoid receptors by dexamethasone [13]. Increased cardiac parasympathetic activity was also noted in CS patients when compared to normal subjects but the etiopathogenesis of this alteration remains unknown [14]. In concordance with the previous reports, our patient was normotensive and had a remarkable recovery in cardiac functions after remission of CD.

Diabetic cardiomyopathy (DCM) may also cause systolic dysfunction but it characteristically develops as a complication of longstanding diabetes and systolic dysfunction is reversible in only early stages of disease. Cardiac hypertrophy is the hallmark finding of DCM on echocardiography [15]. These data do not support DCM as possible diagnosis for our case.

## 4. Conclusions

In summary our experience with this patient supports that hypercortisolism per se had a direct influence on cardiac function, independent of hypertension and left ventricle hypertrophy. Furthermore Cushing's syndrome must be kept in mind as a reversible cause of dilated cardiomyopathy. Surgery must be planned as soon as possible after amelioration of symptoms for patients with cardiac complications.

## Figures and Tables

**Table 1:** Laboratory investigation results.

	Reference range	Presentation	Third month of ketoconazole	After surgery
24-hour urine free cortisol (nmol/d)	19.5–115	138	184	100
ACTH (8 a.m.) (pg/mL)	7.2–63.3	80.1	72	8.1
Cortisol (8 a.m.) (*μ*g/dL)	6.7–22.6	20.2	12.8	1.05
Cortisol (11 p.m.) (*μ*g/dL)	<10	16.3	11.2	3.2

**Table 2:** Echocardiography measurements at presentation and six months after surgery.

	Reference range	Presentation	Postsurgical sixth month
LVEDD	<3.2 cm/m^2^	3.77	3.11
LVESD	<1.9 cm/m^2^	2.91	1.7
EF	50–70%	20	51
IVS	0.8–1.1 cm	1.0	1.0
PW	0.8–1.1 cm	1.0	1.0
